# PIWI Expression and Function in Cancer

**DOI:** 10.3389/fgene.2012.00204

**Published:** 2012-10-16

**Authors:** Ryusuke Suzuki, Shozo Honda, Yohei Kirino

**Affiliations:** ^1^Department of Biomedical Sciences, Samuel Oschin Comprehensive Cancer Institute, Cedars-Sinai Medical CenterLos Angeles, CA, USA

**Keywords:** PIWI, piRNA, cancer, AGO, miRNA, Argonaute family proteins, small regulatory RNAs, non-coding RNAs

## Abstract

PIWI proteins, a subclade of the Argonaute family proteins, are expressed predominantly in the germline and bind to PIWI-interacting RNAs (piRNAs), which are 25–31 nucleotides in length. The PIWI/piRNA pathway plays critical roles in germline development by regulating transposons and other targets to maintain genome integrity. While the functions of PIWI in the germline have been extensively investigated, recent studies have accumulated evidence that the human PIWI proteins, HIWI and HILI, are aberrantly expressed in a variety of cancers. This review summarizes our knowledge of PIWI expression in cancer and discusses its possible role in tumorigenesis.

## Argonaute Family Proteins and Small Regulatory RNAs

In recent years, it has become increasingly apparent that many non-protein-coding regions of the genome are transcribed, and that these non-coding RNAs play crucial roles in normal biological processes and human diseases (Esteller, 2011). The functional significance of non-coding RNAs is particularly evident for small regulatory RNAs, which direct highly specific regulation of gene expression by complementary recognition of their RNA targets. To date, three major classes of small regulatory RNAs have been identified: microRNAs (miRNAs), short-interfering RNAs (siRNAs), and PIWI-interacting RNAs (piRNAs; Farazi et al., 2008; Ghildiyal and Zamore, 2009; Kim et al., 2009). The defining features of small regulatory RNAs are their short lengths of 20–31 nucleotides (nt), and their interaction with Argonaute family proteins to form effector ribonucleoprotein complexes. Argonaute family proteins are well-conserved proteins of approximately 95 kDa and are defined by two major protein motifs: the PAZ domain, a single-stranded nucleic acid-binding motif, and the PIWI domain containing an RNase H fold (Carmell et al., 2002; Parker and Barford, 2006). Based on amino acid sequence similarities, Argonaute family proteins can be divided into two subclades: AGO, named after its founding member in *Arabidopsis thaliana*, and PIWI, named after the *Drosophila* protein PIWI (P-element induced wimpy testis; Carmell et al., 2002).

AGO proteins are ubiquitously expressed in all the tissues and bind to miRNAs and siRNAs that are 20–23 nt in length (Bartel, 2004; Farazi et al., 2008; Liu et al., 2008; Ghildiyal and Zamore, 2009; Kim et al., 2009; Table 1). miRNAs, the best-studied class of small regulatory RNAs, are produced from stem-loop hairpin-structured primary miRNAs (pri-miRNAs), which are processed in the nucleus by the ribonuclease Drosha. The resultant precursor miRNAs (pre-miRNAs) are exported from the nucleus and cleaved in the cytoplasm by a Dicer endonuclease to yield mature miRNAs of approximately 22 nt in length. These miRNAs bind to AGO proteins and repress target mRNA expression by recognizing complementary sequences in the mRNAs which are generally located in the 3 ′-UTR. Imperfect miRNA base-pairing with target mRNAs appears to induce translational silencing, whereas perfect base-pairing triggers exonucleolytic decay of the target mRNAs (Pillai et al., 2007; Liu et al., 2008). The human genome encodes over 1000 miRNAs (Bentwich et al., 2005; Griffiths-Jones et al., 2008), which are estimated to regulate the expression of more than 60% of protein-coding genes (Friedman et al., 2009). Therefore, miRNAs constitute one of the most abundant classes of gene expression regulators and have a tremendous impact on shaping transcriptomes of eukaryotic organisms. siRNAs, which are widely used to experimentally manipulate gene expression, are processed from double-stranded RNA precursors by Dicer. The resulting mature siRNAs bind to AGO proteins, and *in vivo*, the siRNA pathway destabilizes viral RNA to limit virus infectivity and is also involved in transposon regulation (Wang et al., 2006; Farazi et al., 2008; Ghildiyal and Zamore, 2009; Kim et al., 2009).

**Table 1:** **Argonaute family proteins**.

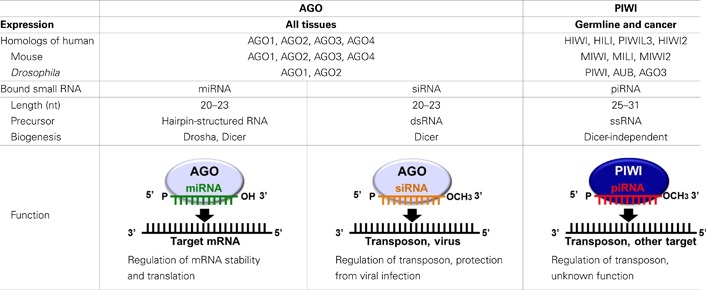

## PIWI and piRNA: A Small Regulatory RNA Pathway in the Germline

In contrast to the ubiquitous expression of AGO proteins, the expression of PIWI proteins, the other subclade of the Argonaute family, is restricted to germline cells. PIWI was first identified in a genetic screen for mutants that affect asymmetric division of stem cells in the *Drosophila* germline (Lin and Spradling, 1997; Cox et al., 1998). The early studies on the *piwi* mutant demonstrated that *Drosophila* PIWI is essential for gametogenesis and is a key regulator of female germline stem cells (Cox et al., 1998, 2000). The PIWI protein family is highly conserved in a wide variety of organisms. Four PIWI proteins are expressed in humans: PIWIL1/HIWI, PIWIL2/HILI, PIWIL3, and PIWIL4/HIWI2 (Sasaki et al., 2003). Three PIWI proteins are expressed in mice: MIWI, MILI, and MIWI2 (Kuramochi-Miyagawa et al., 2001; Deng and Lin, 2002; Carmell et al., 2007). Three PIWI proteins are also expressed in *Drosophila*: PIWI, Aubergine (AUB), and AGO3 (Lin and Spradling, 1997; Cox et al., 1998; Harris and MacDonald, 2001; Brennecke et al., 2007; Gunawardane et al., 2007; Table 1). PIWI mutations in mice, *Drosophila*, and zebrafish commonly cause defects in gametogenesis (Schupbach and Wieschaus, 1991; Cox et al., 1998, 2000; Deng and Lin, 2002; Kuramochi-Miyagawa et al., 2004; Brennecke et al., 2007; Carmell et al., 2007; Chen et al., 2007; Gunawardane et al., 2007; Houwing et al., 2007, 2008), indicating evolutionarily conserved essential roles for PIWI proteins in germline development.

Since 2006, the small RNAs bound to PIWI proteins have been purified and identified in mice (Aravin et al., 2006, 2007, 2008; Girard et al., 2006; Grivna et al., 2006a; Watanabe et al., 2006; Kuramochi-Miyagawa et al., 2008), rats (Lau et al., 2006), *Xenopus* (Armisen et al., 2009; Kirino et al., 2009; Wilczynska et al., 2009), zebrafish (Houwing et al., 2007, 2008), *Drosophila* (Saito et al., 2006; Vagin et al., 2006; Brennecke et al., 2007; Gunawardane et al., 2007), silkworm (Kawaoka et al., 2008), and *C. elegans* (Ruby et al., 2006; Batista et al., 2008). These interacting RNAs are 25–31 nt in length, which are longer than miRNAs and siRNAs by several bases, and termed piRNAs (Klattenhoff and Theurkauf, 2008; Siomi et al., 2011). The molecular mechanism and involved factors for piRNA biogenesis and function remain elusive. piRNAs are a highly complex mix of sequences, with tens of thousands of distinct piRNA sequences, derived from defined genomic regions called piRNA clusters. The main function of piRNAs is to silence transposable elements, and in *Drosophila* ovaries, the vast majority of piRNAs appear to be derived from a limited number of pericentromeric and telomeric sites that are enriched for retrotransposon sequences (Klattenhoff and Theurkauf, 2008; Malone and Hannon, 2009; Siomi et al., 2011). Unlike the other classes of small regulatory RNAs, piRNAs are believed to be generated from single-stranded RNA transcripts by a Dicer-independent mechanism. piRNAs have a preference for uridine at their 5′-ends, and have a HEN1 methyltransferase-catalyzed 2′-*O*-methyl ribose modification at their 3′-ends (Horwich et al., 2007; Kirino and Mourelatos, 2007a,b; Ohara et al., 2007; Saito et al., 2007). Several other factors have been suggested to be involved in piRNA biogenesis, including Armitage, Zucchini, and Squash in *Drosophila* (Vagin et al., 2006; Pane et al., 2007), and MVH, MitoPLD, and SUN1 in mice (Chi et al., 2009; Kuramochi-Miyagawa et al., 2010; Huang et al., 2011; Watanabe et al., 2011). PIWI proteins contain evolutionarily conserved symmetrical-dimethylarginines (sDMAs), which are synthesized by the methyltransferase PRMT5 (Kirino et al., 2009; Vagin et al., 2009). Several members of the TUDOR domain-containing protein family, which specifically recognizes sDMAs such as Spindle-E, Tudor, Krimper, and Tejas in *Drosophila* (Vagin et al., 2006; Lim and Kai, 2007; Nishida et al., 2009; Kirino et al., 2010; Patil and Kai, 2010) and TDRD1-9 in mice (Chen et al., 2009; Kojima et al., 2009; Reuter et al., 2009; Shoji et al., 2009; Vagin et al., 2009; Wang et al., 2009; Kirino et al., 2010), have recently received attention for their PIWI interactions and functional involvement in piRNA biogenesis and function (Siomi et al., 2010).

## PIWI Expression in Cancer

Gene expression in cancer cells and tissues is known to be controlled by a wide array of regulatory molecules including small regulatory RNAs. Among the three major classes of small regulatory RNAs, miRNAs have been most extensively studied in cancer (Li et al., 2010; Farazi et al., 2011). Precise control of miRNAs is crucial for keeping cells in normal physiological states, and the dysregulation of miRNAs has been reported to lead to oncogenesis. When their down-regulation leads to tumor formation, miRNAs act as tumor suppressors; when their overexpression leads to tumor formation, miRNAs can be regarded as oncogenes (Calin et al., 2004; Iorio et al., 2005; Johnson et al., 2005; Akao et al., 2006; Sarhadi et al., 2006; Voorhoeve et al., 2006; Yanaihara et al., 2006; Lee and Dutta, 2007; Sampson et al., 2007). Thus, miRNAs have become one of the key players in oncogenesis, and have attracted a great deal of attention as potential biomarkers for diagnosis as well as potential targets for therapeutic manipulation. Despite the well-known roles of miRNAs in oncogenesis, PIWI proteins and piRNAs have not extensively been studied in cancer.

Cancer cells and germ cells, as well as stem cells, share several characteristics such as rapid proliferation and virtually infinite self-renewal. Therefore, it is not surprising that germline factors would also be involved in oncogenesis; germline-specific factors are becoming a focus of cancer research. A group of molecules called cancer/testis antigens (CTAs) have been receiving increased attention. CTAs are a category of tumor antigens whose normal expression is restricted to male germ cells in the testis (Simpson et al., 2005; Costa et al., 2007; Caballero and Chen, 2009; Cheng et al., 2011). CTAs are regarded as potential immunotherapeutic targets because of their restricted expression and therefore, presumably reduced side effects. Using a loss-of-function approach to search for germline factors in *Drosophila* that are responsible for the growth of malignant brain tumors, Janic et al. (2010) demonstrated that two PIWI proteins, PIWI and AUB, contribute to tumor growth.

While miRNA profiles in cancer have been extensively characterized, PIWI proteins are relatively new players in cancer research, and most studies of PIWI protein expression in human cancers have been published only recently (see Table 2). The first report of PIWI expression in cancer was in seminomas, a cancer of male germ cells (Qiao et al., 2002). HIWI was detected in seminomas, but not in non-seminomas, spermatocytic seminomas, or testicular tumors originating from somatic cells such as Sertoli cells and Leydig cells (Qiao et al., 2002). HILI was also detected in seminomas, and induction of ectopic HILI expression in NIH3T3 cells, a mouse cell line, revealed that HILI is related to cell growth, adhesion, and apoptosis (Lee et al., 2006). These reports on seminomas were followed by reports on a wide variety of cancers (Table 2). HILI was detected in breast cancer (Lee et al., 2010; Liu et al., 2010a) and shown to suppress apoptosis of cancer cells (Lee et al., 2010). Cervical cancer cells were reported to express HIWI (Liu et al., 2010b) and HILI (He et al., 2010; Lu et al., 2012). Interestingly, the expression of both HIWI and HILI showed correlation with human papillomavirus infection (He et al., 2010; Liu et al., 2010b). Elevated HIWI expression was shown to be associated with cancer invasion, but not with patient age or histological grade (Liu et al., 2010b). Furthermore, in cervical cancer, HILI was shown to inhibit p53, a tumor suppressor, and to repress apoptotic cell death of cancer cells (Lu et al., 2012).

**Table 2:** **PIWI expression in human cancers**.

Disease	Material	PIWI	Method	Reference
Breast cancer	Tissue, MDA-MB-231	HILI	RT-PCR, RNA array, WB, IC	Lee et al. (2010)
Breast cancer	Tissue	HILI	IHC	Liu et al. (2010a)
Breast, cervical, and other cancers	MDA-MB-231, MDA-MB-468, MCF-7, HeLa, THP-1, CCRF, Jurkat, H9, Raji, Daudi, HEL, Dami, HL-60, K562, PBL985, HCT-8, 3B11, CaoV3, CaCo, HT-29, SW480, Huh7, CT26CL25, Hey1B, SW872, H1299, C8161, HepG2, INS-1, LL2, N2a	HILI, PL2L50, PL2L60, PL2L80	RT-PCR, WB, IHC	Ye et al. (2010)
Cervical cancer	Tissue	HIWI	IHC	Liu et al. (2010b)
Cervical cancer	Tissue	HILI	IHC	He et al. (2010)
Cervical cancer	HeLa	HILI	WB	Lu et al. (2012)
Colon cancer	Tissue	HIWI	IHC	Liu et al. (2012)
Colorectal and other cancers	Human tissue, 823, AGS, N87, GES1, E30, E70, E140, E180, E410, HepG2, 7402, 7721, YES2, T12, LoVo, CL187, HT-29, RKO, SW480, HCT116, PG, GLC82, H446, H460, H1299, A549	HIWI	WB, IHC	Zeng et al. (2011)
Endometrial cancer	Tissue	HIWI	IHC	Liu et al. (2010c)
Esophageal cancer	Tissue, KYSE70, KYSE140, KYSE450	HIWI	WB, IC, IHC	He et al. (2009)
Gastric cancer	Tissue	HIWI, HILI, PIWIL3, HIWI2	IHC	Wang et al. (2012)
Gastric cancer	Tissue, AGS, NCI-N87, SNU-1, SNU-5, SNU-16	HIWI	RT-PCR, IHC, WB	Liu et al. (2006)
Glioma	Tissue, U251, U87, LN229	HIWI	RT-PCR, WB, IHC	Sun et al. (2011)
Liver cancer	Tissue, HepG2, SMMC7721, MHCC97L, MHCC97H, HCCLM3	HIWI	qRT-PCR, WB, IHC	Zhao et al. (2012)
Ovarian cancer	A2780, CP70, CDDP, MCP2, MCP3, MCP8, 2008, 2008C13	HILI	WB	Wang et al. (2011)
Pancreatic cancer	Tissue	HIWI	qRT-PCR, IHC	Grochola et al. (2008)
Sarcoma	Tissue	HIWI	qRT-PCR	Taubert et al. (2007)
Sarcoma	Tissue, MFH	HIWI	IHC	Siddiqi et al. (2012)
Seminoma	Tissue	HIWI	qRT-PCR	Qiao et al. (2002)
Seminoma and other cancers	Tissue, MDA-MB-231	HILI	RT-PCR, IC, IHC	Lee et al. (2006)

There are additional clinical reports suggesting a potential use for PIWI expression in cancer prognosis. In gliomas, the expression level of HIWI was positively correlated with tumor grade, and patients with high HIWI expression had poorer clinical outcomes (Sun et al., 2011). In pancreatic cancer, patients with an altered level of HIWI mRNA had an increased risk of tumor-related death (Grochola et al., 2008). Among colon cancer patients without lymph node metastasis, those with HIWI-positive tumors had a significantly lower survival rate than those with HIWI-negative tumors, according to Kaplan–Meier analysis (Liu et al., 2012). Furthermore, among patients with colorectal cancer, those with HIWI expression in adjacent non-cancerous tissue had lower survival rates than patients without HIWI expression (Zeng et al., 2011). Among patients with early stage colorectal cancer, HIWI expression was negatively correlated with survival time (Zeng et al., 2011). HIWI has also been detected in endometrial cancer, esophageal cancer, and liver cancer. HIWI expression in endometrial cancer was not associated with clinical pathological features (Liu et al., 2010c); however, in esophageal cancer, it was positively correlated with histological grade and T stage, and was related to poor clinical outcome when the expression was observed in the cytoplasm (He et al., 2009). HIWI expression in liver cancer was positively correlated with tumor size and metastasis, and negatively correlated with survival rates (Zhao et al., 2012). PIWI proteins have been detected in gastric cancer as well (Liu et al., 2006; Wang et al., 2012). The expression of HIWI, HILI, PIWIL3, and HIWI2 were positively correlated with T stage, lymph node metastasis, and clinical TNM, and patients with higher expression had shorter survival times (Wang et al., 2012). Moreover, HIWI has been shown to be an independent prognostic factor in gastric cancer, according to multivariate analyses by Cox’s proportional hazard model (Wang et al., 2012). A more basic study of gastric cancer revealed a correlation between the expression of HIWI and Ki67, a proliferation marker (Liu et al., 2006). The suppression of HIWI caused cell cycle arrest in the G2/M phase and inhibited the growth of gastric cancer cells (Liu et al., 2006). HIWI was reported to be overexpressed in sarcoma, and its increased expression correlated with grade (Siddiqi et al., 2012). HIWI was shown to be a negative prognostic factor for sarcoma patients (Taubert et al., 2007). Lastly, HILI expression has been observed in both ovarian cancer patient tissues (Lee et al., 2006) and cell lines (Wang et al., 2011).

It is noteworthy that HILI (PIWIL2) has been shown to have multiple variant forms. PIWIL2-like (PL2L) proteins have been identified in a wide variety of cancers (Ye et al., 2010). One variant, PL2L60, was found in all human cancer cell lines tested, and its expression was associated with nuclear expression of NF-κB, whose incorrect regulation has been linked to oncogenesis (Perkins, 2012). It is also noteworthy that there have so far been no reports that convincingly demonstrate the presence of piRNAs in cancer. Presumably, many factors, known or unknown, are required for the complex process of piRNA biogenesis and expression in cancer cells. However, there may exist a pathway for PIWI protein function without guidance by piRNAs. In fact, a recent study demonstrated that MIWI directly interacts with and stabilizes mRNAs without piRNAs as guides (Vourekas et al., 2012). Further studies are urgently needed to determine whether PIWI proteins interact with piRNAs in cancer and whether PIWI proteins interact with other RNA species.

## How is PIWI Involved in Tumorigenesis?

As described above, there have been many studies demonstrating PIWI expression in a wide variety of cancers. Some of these are clinical reports utilizing precious patient samples, and it is undoubtedly worth considering the future use of PIWI proteins as potential therapeutic targets. However, in spite of the growing attention focused on PIWI proteins, very few studies have carefully examined the molecular mechanisms by which PIWI proteins contribute to tumorigenesis or function in cancer cells. Therefore, we must rely on studies of normal germ cells for hypothesizing the roles of PIWI proteins in cancer, as described below.

Firstly, the overexpression of PIWI proteins may contribute to tumorigenesis by transcriptionally silencing tumor-suppressing genes through epigenetic mechanisms. In mice, both *mili* and *miwi2* mutants fail to establish *de novo* DNA methylation of transposon sequences, which is required for transcriptional silencing of transposons in the genome, suggesting that MILI and MIWI2 guide the DNA methylation machinery to transposon loci (Aravin et al., 2007, 2008; Kuramochi-Miyagawa et al., 2008). Since MIWI2 with antisense sequences of transposons stays in the nucleus during the short period of embryogenesis when *de novo* DNA methylation occurs in the male germline, it has been speculated that piRNAs function as a guide for directing transposon-specific DNA methylation. In *Drosophila*, PIWI is localized in the nucleus, and its nuclear localization is essential for its function in transposon silencing (Cox et al., 1998; Saito et al., 2006, 2010). It has also been reported that *Drosophila* PIWI interacts with heterochromatin protein 1a (HP1a) and directs HP1a localization to heterochromatin formation (Pal-Bhadra et al., 2004; Brower-Toland et al., 2007). Additionally, PIWI co-localizes with Polycomb group proteins, suggesting its involvement in chromatin-dependent mechanisms (Grimaud et al., 2006). PIWI can also function as an epigenetic activator by promoting euchromatic histone modifications in heterochromatin (Yin and Lin, 2007). Consistent with these observations on epigenetic functions of PIWI/piRNA complexes in the germline, Siddiqi et al. (2012) recently reported that HIWI expression is associated with DNA methylation in sarcoma, and that down-regulation of HIWI reduces global DNA methylation and limits tumorigenesis.

Secondly, analogous with the AGO/miRNA complexes that induce translational silencing of target mRNAs, PIWI proteins may affect the post-transcriptional regulation of oncogenes and tumor suppressor genes. Consistent with this speculation, in mice, MIWI binds to mRNAs, as well as to piRNAs, in the polysome (ribosome cluster) fraction and also associates with mRNA cap-binding complexes (Grivna et al., 2006a,b). Since *miwi* null mice showed down-regulation of the mRNAs normally complexed with MIWI (Deng and Lin, 2002), MIWI positively regulates the stability and probably the translation of its target mRNAs. MILI forms a complex with eIF3a and is also associated with mRNA cap-binding complexes (Unhavaithaya et al., 2009). Although the *mili* mutation had no effect on the cellular mRNA level, it reduced the rate of protein synthesis in premature testicular seminiferous tubules, suggesting a positive role for MILI in translational regulation (Unhavaithaya et al., 2009). Similarly, *Drosophila* PIWI proteins have been shown to positively regulate translation in early embryos. PIWI overexpression enhances the expression of Oskar and Vasa (Megosh et al., 2006). AUB does not affect the levels of oskar mRNAs, but enhances their translation (Wilson et al., 1996). In contrast, AUB and piRNAs can act as a post-transcriptional negative regulator by promoting deadenylation and decay of maternal mRNAs in embryos (Rouget et al., 2010).

Thirdly, PIWI proteins may be involved in genomic instability, one of the most common occurrences in cancer. Genomic instability is attributable to an extra copy of genomic DNA or a chromosome, chromosomal translocation, chromosomal deletion, or single-stranded or double-stranded breaks in genomic DNA (Cassidy and Venkitaraman, 2012; Lord and Ashworth, 2012). It has been demonstrated that LINE1, a known target retrotransposon of the PIWI/piRNA pathway in the germline, contributes to DNA repair through its integration into DNA lesions (Morrish et al., 2002, 2007; Zingler et al., 2005). This implies that PIWI proteins in cancer may cause genomic instability by suppressing the expression of such transposons. However, mice and *Drosophila* deficient for PIWI proteins accumulate γ-H2Av foci, a sign of double-stranded DNA breaks, suggesting a positive contribution of PIWI proteins to the repair of DNA damage (Kuramochi-Miyagawa et al., 2004; Carmell et al., 2007; Klattenhoff et al., 2007). A putative positive role for PIWI in DNA repair is supported by the observation that RIWI, a rat PIWI protein, forms a complex with RecQ1 (Lau et al., 2006), which has a highly conserved role in the repair of double-stranded DNA breaks (Hunter, 2008). In an ovarian cancer cell line, HILI has been reported to repair cisplatin–induced DNA damage and help cancer cells survive platinum-based chemotherapy (Wang et al., 2011). Therefore, if and how PIWI proteins are involved in DNA repair, and therefore in genomic instability, remains controversial.

Fourthly, PIWI proteins may promote cell proliferation in cancer and cause aneuploidy during mitosis. Pek and Kai (2011) have recently reported an abolished localization of Vasa in mitotic chromosomes, defective chromosomal condensation and segregation, and delayed cell division in the germline of an *aub Drosophila* mutant. Vasa is a conserved germline DEAD-box RNA helicase that plays diverse roles in the regulation of mRNA translation, germline differentiation, germ granule assembly, and piRNA-mediated transposon silencing (Liang et al., 1994; Styhler et al., 1998; Vagin et al., 2004; Malone et al., 2009; Kuramochi-Miyagawa et al., 2010). AUB mediates the localization of Vasa in the vicinity of mitotic chromosomes; Vasa recruits condensin I and promotes robust chromosomal condensation and segregation (Pek and Kai, 2011). This raises the intriguing possibility that PIWI proteins and piRNAs might regulate cell division in cancer cells.

## Concluding Remarks

A growing number of reports have revealed the aberrant expression of PIWI proteins in various cancers, and it appears highly plausible that PIWI proteins are involved in tumorigenesis. However, data for elucidating the detailed molecular role of PIWI proteins in tumorigenesis is very limited, in part because a considerable portion of the experimental results is derived from studies of patient samples, with limited opportunities for experimental manipulation. Further basic studies with more manipulable materials, such as cell lines and experimental animals, are urgently needed to address the possibility of PIWI as a therapeutic target. An immediate focus is to identify PIWI protein-associating RNAs to determine whether piRNAs and/or other RNA species specifically interact with PIWI proteins in cancer cells. It is also imperative to investigate how PIWI proteins are involved in the biological functioning of cancer cells, such as transposon silencing, transcriptional or post-transcriptional regulation, DNA repair, and chromosome condensation and segregation, and to determine the roles of PIWI proteins in tumorigenesis. Such investigations will significantly advance our understanding of tumorigenesis and may lead to novel therapeutic applications targeting PIWI proteins and their molecular functions.

## Conflict of Interest Statement

The authors declare that the research was conducted in the absence of any commercial or financial relationships that could be construed as a potential conflict of interest.

## References

[B1] Akao Y. Nakagawa Y. Naoe T. (2006). let-7 microRNA functions as a potential growth suppressor in human colon cancer cells. Biol. Pharm. Bull. 29, 903–906 10.1248/bpb.29.903 16651716

[B2] Aravin A. Gaidatzis D. Pfeffer S. Lagos-Quintana M. Landgraf P. Iovino N. (2006). A novel class of small RNAs bind to MILI protein in mouse testes. Nature 442, 203–207 16751777 10.1038/nature04916

[B3] Aravin A. A. Sachidanandam R. Bourc’his D. Schaefer C. Pezic D. Toth K. F. (2008). A piRNA pathway primed by individual transposons is linked to de novo DNA methylation in mice. Mol. Cell 31, 785–799 10.1016/j.molcel.2008.09.003 18922463 PMC2730041

[B4] Aravin A. A. Sachidanandam R. Girard A. Fejes-Toth K. Hannon G. J. (2007). Developmentally regulated piRNA clusters implicate MILI in transposon control. Science 316, 744–747 10.1126/science.1142612 17446352

[B5] Armisen J. Gilchrist M. J. Wilczynska A. Standart N. Miska E. A. (2009). Abundant and dynamically expressed miRNAs, piRNAs, and other small RNAs in the vertebrate Xenopus tropicalis. Genome Res. 19, 1766–1775 10.1101/gr.093054.109 19628731 PMC2765267

[B6] Bartel D. P. (2004). MicroRNAs: genomics, biogenesis, mechanism, and function. Cell 116, 281–297 10.1016/S0092-8674(04)00045-5 14744438

[B7] Batista P. J. Ruby J. G. Claycomb J. M. Chiang R. Fahlgren N. Kasschau K. D. (2008). PRG-1 and 21U-RNAs interact to form the piRNA complex required for fertility in C. elegans. Mol. Cell 31, 67–78 10.1016/j.molcel.2008.06.002 18571452 PMC2570341

[B8] Bentwich I. Avniel A. Karov Y. Aharonov R. Gilad S. Barad O. (2005). Identification of hundreds of conserved and nonconserved human microRNAs. Nat. Genet. 37, 766–770 10.1038/ng1590 15965474

[B9] Brennecke J. Aravin A. A. Stark A. Dus M. Kellis M. Sachidanandam R. (2007). Discrete small RNA-generating loci as master regulators of transposon activity in Drosophila. Cell 128, 1089–1103 10.1016/j.cell.2007.01.043 17346786

[B10] Brower-Toland B. Findley S. D. Jiang L. Liu L. Yin H. Dus M. (2007). Drosophila PIWI associates with chromatin and interacts directly with HP1a. Genes Dev. 21, 2300–2311 10.1101/gad.1564307 17875665 PMC1973144

[B11] Caballero O. L. Chen Y. T. (2009). Cancer/testis (CT) antigens: potential targets for immunotherapy. Cancer Sci. 100, 2014–2021 10.1111/j.1349-7006.2009.01303.x 19719775 PMC11158245

[B12] Calin G. A. Sevignani C. Dumitru C. D. Hyslop T. Noch E. Yendamuri S. (2004). Human microRNA genes are frequently located at fragile sites and genomic regions involved in cancers. Proc. Natl. Acad. Sci. U.S.A. 101, 2999–3004 10.1073/pnas.0404432101 14973191 PMC365734

[B13] Carmell M. A. Girard A. Van De Kant H. J. Bourc’his D. Bestor T. H. De Rooij D. G. (2007). MIWI2 is essential for spermatogenesis and repression of transposons in the mouse male germline. Dev. Cell 12, 503–514 10.1016/j.devcel.2007.03.001 17395546

[B14] Carmell M. A. Xuan Z. Zhang M. Q. Hannon G. J. (2002). The Argonaute family: tentacles that reach into RNAi, developmental control, stem cell maintenance, and tumorigenesis. Genes Dev. 16, 2733–2742 10.1101/gad.1026102 12414724

[B15] Cassidy L. D. Venkitaraman A. R. (2012). Genome instability mechanisms and the structure of cancer genomes. Curr. Opin. Genet. Dev. 22, 10–13 10.1016/j.gde.2012.02.003 22366532

[B16] Chen C. Jin J. James D. A. Adams-Cioaba M. A. Park J. G. Guo Y. (2009). Mouse Piwi interactome identifies binding mechanism of Tdrkh Tudor domain to arginine methylated Miwi. Proc. Natl. Acad. Sci. U.S.A. 106, 20336–20341 10.1073/pnas.0903497106 19918066 PMC2787181

[B17] Chen Y. Pane A. Schupbach T. (2007). Cutoff and aubergine mutations result in retrotransposon upregulation and checkpoint activation in Drosophila. Curr. Biol. 17, 637–642 10.1016/j.cub.2007.01.031 17363252 PMC1905832

[B18] Cheng Y. H. Wong E. W. Cheng C. Y. (2011). Cancer/testis (CT) antigens, carcinogenesis and spermatogenesis. Spermatogenesis 1, 209–220 10.4161/spmg.1.3.17076 22319669 PMC3271663

[B19] Chi Y. H. Cheng L. I. Myers T. Ward J. M. Williams E. Su Q. (2009). Requirement for Sun1 in the expression of meiotic reproductive genes and piRNA. Development 136, 965–973 10.1242/dev.029868 19211677 PMC2727561

[B20] Costa F. F. Le Blanc K. Brodin B. (2007). Concise review: cancer/testis antigens, stem cells, and cancer. Stem Cells 25, 707–711 10.1634/stemcells.2006-0469 17138959

[B21] Cox D. N. Chao A. Baker J. Chang L. Qiao D. Lin H. (1998). A novel class of evolutionarily conserved genes defined by piwi are essential for stem cell self-renewal. Genes Dev. 12, 3715–3727 10.1101/gad.12.23.3715 9851978 PMC317255

[B22] Cox D. N. Chao A. Lin H. (2000). Piwi encodes a nucleoplasmic factor whose activity modulates the number and division rate of germline stem cells. Development 127, 503–514 10631171 10.1242/dev.127.3.503

[B23] Deng W. Lin H. (2002). Miwi, a murine homolog of piwi, encodes a cytoplasmic protein essential for spermatogenesis. Dev. Cell 2, 819–830 10.1016/S1534-5807(02)00165-X 12062093

[B24] Esteller M. (2011). Non-coding RNAs in human disease. Nat. Rev. Genet. 12, 861–874 10.1038/ni.2073 22094949

[B25] Farazi T. A. Juranek S. A. Tuschl T. (2008). The growing catalog of small RNAs and their association with distinct Argonaute/Piwi family members. Development 135, 1201–1214 10.1242/dev.005629 18287206

[B26] Farazi T. A. Spitzer J. I. Morozov P. Tuschl T. (2011). miRNAs in human cancer. J. Pathol. 223, 102–115 10.1002/path.2806 21125669 PMC3069496

[B27] Friedman R. C. Farh K. K. Burge C. B. Bartel D. P. (2009). Most mammalian mRNAs are conserved targets of microRNAs. Genome Res. 19, 92–105 10.1101/gr.082701.108 18955434 PMC2612969

[B28] Ghildiyal M. Zamore P. D. (2009). Small silencing RNAs: an expanding universe. Nat. Rev. Genet. 10, 94–108 10.1038/nrg2504 19148191 PMC2724769

[B29] Girard A. Sachidanandam R. Hannon G. J. Carmell M. A. (2006). A germline-specific class of small RNAs binds mammalian Piwi proteins. Nature 442, 199–202 16751776 10.1038/nature04917

[B30] Griffiths-Jones S. Saini H. K. Van Dongen S. Enright A. J. (2008). miRBase: tools for microRNA genomics. Nucleic Acids Res. 36, D154–D158 10.1093/nar/gkm952 17991681 PMC2238936

[B31] Grimaud C. Bantignies F. Pal-Bhadra M. Ghana P. Bhadra U. Cavalli G. (2006). RNAi components are required for nuclear clustering of Polycomb group response elements. Cell 124, 957–971 10.1016/j.cell.2006.01.036 16530043

[B32] Grivna S. T. Beyret E. Wang Z. Lin H. (2006a). A novel class of small RNAs in mouse spermatogenic cells. Genes Dev. 20, 1709–1714 10.1101/gad.1434406 16766680 PMC1522066

[B33] Grivna S. T. Pyhtila B. Lin H. (2006b). MIWI associates with translational machinery and PIWI-interacting RNAs (piRNAs) in regulating spermatogenesis. Proc. Natl. Acad. Sci. U.S.A. 103, 13415–13420 10.1073/pnas.0605506103 16938833 PMC1569178

[B34] Grochola L. F. Greither T. Taubert H. Moller P. Knippschild U. Udelnow A. (2008). The stem cell-associated Hiwi gene in human adenocarcinoma of the pancreas: expression and risk of tumour-related death. Br. J. Cancer 99, 1083–1088 10.1038/sj.bjc.6604653 18781170 PMC2567072

[B35] Gunawardane L. S. Saito K. Nishida K. M. Miyoshi K. Kawamura Y. Nagami T. (2007). A slicer-mediated mechanism for repeat-associated siRNA 5’ end formation in Drosophila. Science 315, 1587–1590 10.1126/science.1140494 17322028

[B36] Harris A. N. MacDonald P. M. (2001). Aubergine encodes a Drosophila polar granule component required for pole cell formation and related to eIF2C. Development 128, 2823–2832 11526087 10.1242/dev.128.14.2823

[B37] He G. Chen L. Ye Y. Xiao Y. Hua K. Jarjoura D. (2010). Piwil2 expressed in various stages of cervical neoplasia is a potential complementary marker for p16. Am. J. Transl. Res. 2, 156–169 20407605 PMC2855633

[B38] He W. Wang Z. Wang Q. Fan Q. Shou C. Wang J. (2009). Expression of HIWI in human esophageal squamous cell carcinoma is significantly associated with poorer prognosis. BMC Cancer 9, 426 10.1186/1471-2407-9-426 19995427 PMC2801519

[B39] Horwich M. D. Li C. Matranga C. Vagin V. Farley G. Wang P. (2007). The Drosophila RNA methyltransferase, DmHen1, modifies germline piRNAs and single-stranded siRNAs in RISC. Curr. Biol. 17, 1265–1272 10.1016/j.cub.2007.06.030 17604629

[B40] Houwing S. Berezikov E. Ketting R. F. (2008). Zili is required for germ cell differentiation and meiosis in zebrafish. EMBO J. 27, 2702–2711 10.1038/emboj.2008.204 18833190 PMC2572183

[B41] Houwing S. Kamminga L. M. Berezikov E. Cronembold D. Girard A. Van Den Elst H. (2007). A role for Piwi and piRNAs in germ cell maintenance and transposon silencing in Zebrafish. Cell 129, 69–82 10.1016/j.cell.2007.03.026 17418787

[B42] Huang H. Gao Q. Peng X. Choi S. Y. Sarma K. Ren H. (2011). piRNA-associated germline nuage formation and spermatogenesis require MitoPLD profusogenic mitochondrial-surface lipid signaling. Dev. Cell 20, 376–387 10.1016/j.devcel.2011.01.004 21397848 PMC3061402

[B43] Hunter N. (2008). The RecQ DNA helicases: jacks-of-all-trades or master-tradesmen? Cell Res. 18, 328–330 10.1038/cr.2008.33 18311162

[B44] Iorio M. V. Ferracin M. Liu C. G. Veronese A. Spizzo R. Sabbioni S. (2005). MicroRNA gene expression deregulation in human breast cancer. Cancer Res. 65, 7065–7070 10.1158/0008-5472.CAN-05-1783 16103053

[B45] Janic A. Mendizabal L. Llamazares S. Rossell D. Gonzalez C. (2010). Ectopic expression of germline genes drives malignant brain tumor growth in Drosophila. Science 330, 1824–1827 10.1126/science.1195481 21205669

[B46] Johnson S. M. Grosshans H. Shingara J. Byrom M. Jarvis R. Cheng A. (2005). RAS is regulated by the let-7 microRNA family. Cell 120, 635–647 10.1016/j.cell.2005.01.014 15766527

[B47] Kawaoka S. Hayashi N. Katsuma S. Kishino H. Kohara Y. Mita K. (2008). Bombyx small RNAs: genomic defense system against transposons in the silkworm, Bombyx mori. Insect Biochem. Mol. Biol. 38, 1058–1065 10.1016/j.ibmb.2008.03.007 18801438

[B48] Kim V. N. Han J. Siomi M. C. (2009). Biogenesis of small RNAs in animals. Nat. Rev. Mol. Cell Biol. 10, 126–139 10.1038/nrm2632 19165215

[B49] Kirino Y. Kim N. De Planell-Saguer M. Khandros E. Chiorean S. Klein P. S. (2009). Arginine methylation of Piwi proteins catalysed by dPRMT5 is required for Ago3 and Aub stability. Nat. Cell Biol. 11, 652–658 10.1038/ncb1872 19377467 PMC2746449

[B50] Kirino Y. Mourelatos Z. (2007a). The mouse homolog of HEN1 is a potential methylase for Piwi-interacting RNAs. RNA 13, 1397–1401 10.1261/rna.659307 17652135 PMC1950760

[B51] Kirino Y. Mourelatos Z. (2007b). Mouse Piwi-interacting RNAs are 2’-O-methylated at their 3’ termini. Nat. Struct. Mol. Biol. 14, 347–348 10.1038/nsmb1218 17384647

[B52] Kirino Y. Vourekas A. Sayed N. De Lima Alves F. Thomson T. Lasko P. (2010). Arginine methylation of Aubergine mediates Tudor binding and germ plasm localization. RNA 16, 70–78 10.1261/rna.1869710 19926723 PMC2802038

[B53] Klattenhoff C. Bratu D. P. McGinnis-Schultz N. Koppetsch B. S. Cook H. A. Theurkauf W. E. (2007). Drosophila rasiRNA pathway mutations disrupt embryonic axis specification through activation of an ATR/Chk2 DNA damage response. Dev. Cell 12, 45–55 10.1016/j.devcel.2006.12.001 17199040

[B54] Klattenhoff C. Theurkauf W. (2008). Biogenesis and germline functions of piRNAs. Development 135, 3–9 10.1242/dev.006486 18032451

[B55] Kojima K. Kuramochi-Miyagawa S. Chuma S. Tanaka T. Nakatsuji N. Kimura T. (2009). Associations between PIWI proteins and TDRD1/MTR-1 are critical for integrated subcellular localization in murine male germ cells. Genes Cells 14, 1155–1165 10.1111/j.1365-2443.2009.01342.x 19735482

[B56] Kuramochi-Miyagawa S. Kimura T. Ijiri T. W. Isobe T. Asada N. Fujita Y. (2004). Mili, a mammalian member of piwi family gene, is essential for spermatogenesis. Development 131, 839–849 10.1242/dev.00973 14736746

[B57] Kuramochi-Miyagawa S. Kimura T. Yomogida K. Kuroiwa A. Tadokoro Y. Fujita Y. (2001). Two mouse piwi-related genes: miwi and mili. Mech. Dev. 108, 121–133 10.1016/S0925-4773(01)00499-3 11578866

[B58] Kuramochi-Miyagawa S. Watanabe T. Gotoh K. Takamatsu K. Chuma S. Kojima-Kita K. (2010). MVH in piRNA processing and gene silencing of retrotransposons. Genes Dev. 24, 887–892 10.1101/gad.1902110 20439430 PMC2861188

[B59] Kuramochi-Miyagawa S. Watanabe T. Gotoh K. Totoki Y. Toyoda A. Ikawa M. (2008). DNA methylation of retrotransposon genes is regulated by Piwi family members MILI and MIWI2 in murine fetal testes. Genes Dev. 22, 908–917 10.1101/gad.1640708 18381894 PMC2279202

[B60] Lau N. C. Seto A. G. Kim J. Kuramochi-Miyagawa S. Nakano T. Bartel D. P. (2006). Characterization of the piRNA complex from rat testes. Science 313, 363–367 10.1126/science.1130164 16778019

[B61] Lee J. H. Jung C. Javadian-Elyaderani P. Schweyer S. Schutte D. Shoukier M. (2010). Pathways of proliferation and antiapoptosis driven in breast cancer stem cells by stem cell protein piwil2. Cancer Res. 70, 4569–4579 10.1158/0008-5472.SABCS10-P6-10-05 20460541

[B62] Lee J. H. Schutte D. Wulf G. Fuzesi L. Radzun H. J. Schweyer S. (2006). Stem-cell protein Piwil2 is widely expressed in tumors and inhibits apoptosis through activation of Stat3/Bcl-XL pathway. Hum. Mol. Genet. 15, 201–211 10.1093/hmg/ddi430 16377660

[B63] Lee Y. S. Dutta A. (2007). The tumor suppressor microRNA let-7 represses the HMGA2 oncogene. Genes Dev. 21, 1025–1030 10.1101/gad.1518407 17437991 PMC1855228

[B64] Li M. Li J. Ding X. He M. Cheng S. Y. (2010). microRNA and cancer. AAPS J. 12, 309–317 10.1208/s12248-010-9181-5 20422339 PMC2895440

[B65] Liang L. Diehl-Jones W. Lasko P. (1994). Localization of vasa protein to the Drosophila pole plasm is independent of its RNA-binding and helicase activities. Development 120, 1201–1211 8026330 10.1242/dev.120.5.1201

[B66] Lim A. K. Kai T. (2007). Unique germ-line organelle, nuage, functions to repress selfish genetic elements in Drosophila melanogaster. Proc. Natl. Acad. Sci. U.S.A. 104, 6714–6719 10.1073/pnas.0701920104 17428915 PMC1871851

[B67] Lin H. Spradling A. C. (1997). A novel group of pumilio mutations affects the asymmetric division of germline stem cells in the Drosophila ovary. Development 124, 2463–2476 9199372 10.1242/dev.124.12.2463

[B68] Liu C. Qu L. Dong B. Xing X. Ren T. Zeng Y. (2012). Combined phenotype of 4 markers improves prognostic value of patients with colon cancer. Am. J. Med. Sci. 343, 295–302 10.1097/MAJ.0b013e31822b02f4 22261620

[B69] Liu J. J. Shen R. Chen L. Ye Y. He G. Hua K. (2010a). Piwil2 is expressed in various stages of breast cancers and has the potential to be used as a novel biomarker. Int. J. Clin. Exp. Pathol. 3, 328–337 20490325 PMC2872741

[B70] Liu W. K. Jiang X. Y. Zhang Z. X. (2010b). Expression of PSCA, PIWIL1 and TBX2 and its correlation with HPV16 infection in formalin-fixed, paraffin-embedded cervical squamous cell carcinoma specimens. Arch. Virol. 155, 657–663 10.1007/s00705-010-0746-5 20229117

[B71] Liu W. K. Jiang X. Y. Zhang Z. X. (2010c). Expression of PSCA, PIWIL1, and TBX2 in endometrial adenocarcinoma. Onkologie 33, 241–245 10.1159/000319692 20502058

[B72] Liu X. Fortin K. Mourelatos Z. (2008). MicroRNAs: biogenesis and molecular functions. Brain Pathol. 18, 113–121 10.1111/j.1750-3639.2007.00121.x 18226106 PMC8095604

[B73] Liu X. Sun Y. Guo J. Ma H. Li J. Dong B. (2006). Expression of hiwi gene in human gastric cancer was associated with proliferation of cancer cells. Int. J. Cancer 118, 1922–1929 10.1002/ijc.21652 16287078

[B74] Lord C. J. Ashworth A. (2012). The DNA damage response and cancer therapy. Nature 481, 287–294 10.1038/nature10760 22258607

[B75] Lu Y. Zhang K. Li C. Yao Y. Tao D. Liu Y. (2012). Piwil2 suppresses p53 by inducing phosphorylation of signal transducer and activator of transcription 3 in tumor cells. PLoS ONE 7, e30999 10.1371/journal.pone.0030999 22303479 PMC3267750

[B76] Malone C. D. Brennecke J. Dus M. Stark A. McCombie W. R. Sachidanandam R. (2009). Specialized piRNA pathways act in germline and somatic tissues of the Drosophila ovary. Cell 137, 522–535 10.1016/j.cell.2009.03.040 19395010 PMC2882632

[B77] Malone C. D. Hannon G. J. (2009). Small RNAs as guardians of the genome. Cell 136, 656–668 10.1016/j.cell.2009.01.045 19239887 PMC2792755

[B78] Megosh H. B. Cox D. N. Campbell C. Lin H. (2006). The role of PIWI and the miRNA machinery in Drosophila germline determination. Curr. Biol. 16, 1884–1894 10.1016/j.cub.2006.08.051 16949822

[B79] Morrish T. A. Garcia-Perez J. L. Stamato T. D. Taccioli G. E. Sekiguchi J. Moran J. V. (2007). Endonuclease-independent LINE-1 retrotransposition at mammalian telomeres. Nature 446, 208–212 10.1038/nature05560 17344853

[B80] Morrish T. A. Gilbert N. Myers J. S. Vincent B. J. Stamato T. D. Taccioli G. E. (2002). DNA repair mediated by endonuclease-independent LINE-1 retrotransposition. Nat. Genet. 31, 159–165 10.1038/ng898 12006980

[B81] Nishida K. M. Okada T. N. Kawamura T. Mituyama T. Kawamura Y. Inagaki S. (2009). Functional involvement of Tudor and dPRMT5 in the piRNA processing pathway in Drosophila germlines. EMBO J. 28, 3820–3831 10.1038/emboj.2009.365 19959991 PMC2797066

[B82] Ohara T. Sakaguchi Y. Suzuki T. Ueda H. Miyauchi K. (2007). The 3’ termini of mouse Piwi-interacting RNAs are 2’-O-methylated. Nat. Struct. Mol. Biol. 14, 349–350 10.1038/nsmb1220 17384646

[B83] Pal-Bhadra M. Leibovitch B. A. Gandhi S. G. Rao M. Bhadra U. Birchler J. A. (2004). Heterochromatic silencing and HP1 localization in Drosophila are dependent on the RNAi machinery. Science 303, 669–672 10.1126/science.1092653 14752161

[B84] Pane A. Wehr K. Schupbach T. (2007). Zucchini and squash encode two putative nucleases required for rasiRNA production in the Drosophila germline. Dev. Cell 12, 851–862 10.1016/j.devcel.2007.03.022 17543859 PMC1945814

[B85] Parker J. S. Barford D. (2006). Argonaute: a scaffold for the function of short regulatory RNAs. Trends Biochem. Sci. 31, 622–630 10.1016/j.tibs.2006.09.010 17029813

[B86] Patil V. S. Kai T. (2010). Repression of retroelements in Drosophila germline via piRNA pathway by the Tudor domain protein Tejas. Curr. Biol. 20, 724–730 10.1016/j.cub.2010.02.046 20362446

[B87] Pek J. W. Kai T. (2011). A role for vasa in regulating mitotic chromosome condensation in Drosophila. Curr. biol. 21, 39–44 10.1016/j.cub.2010.11.051 21185189

[B88] Perkins N. D. (2012). The diverse and complex roles of NF-kappaB subunits in cancer. Nat. Rev. Cancer 12, 121–132 22257950 10.1038/nrc3204

[B89] Pillai R. S. Bhattacharyya S. N. Filipowicz W. (2007). Repression of protein synthesis by miRNAs: how many mechanisms? Trends Cell Biol. 17, 118–126 10.1016/j.tcb.2006.12.007 17197185

[B90] Qiao D. Zeeman A. M. Deng W. Looijenga L. H. Lin H. (2002). Molecular characterization of hiwi, a human member of the piwi gene family whose overexpression is correlated to seminomas. Oncogene 21, 3988–3999 10.1038/sj.onc.1205505 12037681

[B91] Reuter M. Chuma S. Tanaka T. Franz T. Stark A. Pillai R. S. (2009). Loss of the Mili-interacting Tudor domain-containing protein-1 activates transposons and alters the Mili-associated small RNA profile. Nat. Struct. Mol. Biol. 16, 639–646 10.1038/nsmb.1615 19465913

[B92] Rouget C. Papin C. Boureux A. Meunier A. C. Franco B. Robine N. (2010). Maternal mRNA deadenylation and decay by the piRNA pathway in the early Drosophila embryo. Nature 467, 1128–1132 10.1038/nature09465 20953170 PMC4505748

[B93] Ruby J. G. Jan C. Player C. Axtell M. J. Lee W. Nusbaum C. (2006). Large-scale sequencing reveals 21U-RNAs and additional microRNAs and endogenous siRNAs in C. elegans. Cell 127, 1193–1207 10.1016/j.cell.2006.10.040 17174894

[B94] Saito K. Ishizu H. Komai M. Kotani H. Kawamura Y. Nishida K. M. (2010). Roles for the Yb body components Armitage and Yb in primary piRNA biogenesis in Drosophila. Genes Dev. 24, 2493–2498 10.1101/gad.1989510 20966047 PMC2975925

[B95] Saito K. Nishida K. M. Mori T. Kawamura Y. Miyoshi K. Nagami T. (2006). Specific association of Piwi with rasiRNAs derived from retrotransposon and heterochromatic regions in the Drosophila genome. Genes Dev. 20, 2214–2222 10.1101/gad.1454806 16882972 PMC1553205

[B96] Saito K. Sakaguchi Y. Suzuki T. Siomi H. Siomi M. C. (2007). Pimet, the Drosophila homolog of HEN1, mediates 2’-O-methylation of Piwi- interacting RNAs at their 3’ ends. Genes Dev. 21, 1603–1608 10.1101/gad.1563607 17606638 PMC1899469

[B97] Sampson V. B. Rong N. H. Han J. Yang Q. Aris V. Soteropoulos P. (2007). MicroRNA let-7a down-regulates MYC and reverts MYC-induced growth in Burkitt lymphoma cells. Cancer Res. 67, 9762–9770 10.1158/0008-5472.CAN-07-2462 17942906

[B98] Sarhadi V. K. Wikman H. Salmenkivi K. Kuosma E. Sioris T. Salo J. (2006). Increased expression of high mobility group A proteins in lung cancer. J. Pathol. 209, 206–212 10.1002/path.1960 16521118

[B99] Sasaki T. Shiohama A. Minoshima S. Shimizu N. (2003). Identification of eight members of the Argonaute family in the human genome small star, filled. Genomics 82, 323–330 10.1016/S0888-7543(03)00129-0 12906857

[B100] Schupbach T. Wieschaus E. (1991). Female sterile mutations on the second chromosome of Drosophila melanogaster. II. Mutations blocking oogenesis or altering egg morphology. Genetics 129, 1119–1136 1783295 10.1093/genetics/129.4.1119 PMC1204776

[B101] Shoji M. Tanaka T. Hosokawa M. Reuter M. Stark A. Kato Y. (2009). The TDRD9-MIWI2 complex is essential for piRNA-mediated retrotransposon silencing in the mouse male germline. Dev. Cell 17, 775–787 10.1016/j.devcel.2009.10.012 20059948

[B102] Siddiqi S. Terry M. Matushansky I. (2012). Hiwi mediated tumorigenesis is associated with DNA hypermethylation. PLoS ONE 7, e33711 10.1371/journal.pone.0033711 22438986 PMC3306289

[B103] Simpson A. J. Caballero O. L. Jungbluth A. Chen Y. T. Old L. J. (2005). Cancer/testis antigens, gametogenesis and cancer. Nat. Rev. Cancer 5, 615–625 10.1038/nrc1669 16034368

[B104] Siomi M. C. Mannen T. Siomi H. (2010). How does the royal family of Tudor rule the PIWI-interacting RNA pathway? Genes Dev. 24, 636–646 10.1101/gad.1899210 20360382 PMC2849120

[B105] Siomi M. C. Sato K. Pezic D. Aravin A. A. (2011). PIWI-interacting small RNAs: the vanguard of genome defence. Nat. Rev. Mol. Cell Biol. 12, 246–258 10.1038/nrm3089 21427766

[B106] Styhler S. Nakamura A. Swan A. Suter B. Lasko P. (1998). Vasa is required for GURKEN accumulation in the oocyte, and is involved in oocyte differentiation and germline cyst development. Development 125, 1569–1578 9521895 10.1242/dev.125.9.1569

[B107] Sun G. Wang Y. Sun L. Luo H. Liu N. Fu Z. (2011). Clinical significance of Hiwi gene expression in gliomas. Brain Res. 1373, 183–188 10.1016/j.brainres.2010.11.097 21138738

[B108] Taubert H. Greither T. Kaushal D. Wurl P. Bache M. Bartel F. (2007). Expression of the stem cell self-renewal gene Hiwi and risk of tumour-related death in patients with soft-tissue sarcoma. Oncogene 26, 1098–1100 10.1038/sj.onc.1209880 16953229

[B109] Unhavaithaya Y. Hao Y. Beyret E. Yin H. Kuramochi-Miyagawa S. Nakano T. (2009). MILI, a PIWI-interacting RNA-binding protein, is required for germ line stem cell self-renewal and appears to positively regulate translation. J. Biol. Chem. 284, 6507–6519 10.1074/jbc.M809104200 19114715 PMC2649106

[B110] Vagin V. V. Klenov M. S. Kalmykova A. I. Stolyarenko A. D. Kotelnikov R. N. Gvozdev V. A. (2004). The RNA interference proteins and vasa locus are involved in the silencing of retrotransposons in the female germline of *Drosophila melanogaster*. RNA Biol 1, 54–58 10.4161/rna.1.1.943 17194939

[B111] Vagin V. V. Sigova A. Li C. Seitz H. Gvozdev V. Zamore P. D. (2006). A distinct small RNA pathway silences selfish genetic elements in the germline. Science 313, 320–324 10.1126/science.1129333 16809489

[B112] Vagin V. V. Wohlschlegel J. Qu J. Jonsson Z. Huang X. Chuma S. (2009). Proteomic analysis of murine Piwi proteins reveals a role for arginine methylation in specifying interaction with Tudor family members. Genes Dev. 23, 1749–1762 10.1101/gad.1814809 19584108 PMC2720255

[B113] Voorhoeve P. M. Le Sage C. Schrier M. Gillis A. J. Stoop H. Nagel R. (2006). A genetic screen implicates miRNA-372 and miRNA-373 as oncogenes in testicular germ cell tumors. Cell 124, 1169–1181 10.1016/j.cell.2006.02.037 16564011

[B114] Vourekas A. Zheng Q. Alexiou P. Maragkakis M. Kirino Y. Gregory B. D. (2012). Mili and Miwi target RNA repertoire reveals piRNA biogenesis and function of Miwi in spermiogenesis. Nat. Struct. Mol. Biol. 19, 773–781 10.1038/nsmb.2347 22842725 PMC3414646

[B115] Wang J. Saxe J. P. Tanaka T. Chuma S. Lin H. (2009). Mili interacts with tudor domain-containing protein 1 in regulating spermatogenesis. Curr. Biol. 19, 640–644 10.1016/j.cub.2009.06.020 19345100 PMC2704239

[B116] Wang Q. E. Han C. Milum K. Wani A. A. (2011). Stem cell protein Piwil2 modulates chromatin modifications upon cisplatin treatment. Mutat. Res. 708, 59–68 10.1016/j.mrfmmm.2011.02.001 21310163 PMC3091508

[B117] Wang X. H. Aliyari R. Li W. X. Li H. W. Kim K. Carthew R. (2006). RNA interference directs innate immunity against viruses in adult Drosophila. Science 312, 452–454 10.1126/science.1125694 16556799 PMC1509097

[B118] Wang Y. Liu Y. Shen X. Zhang X. Chen X. Yang C. (2012). The PIWI protein acts as a predictive marker for human gastric cancer. Int. J. Clin. Exp. Pathol. 5, 315–325 22670175 PMC3365820

[B119] Watanabe T. Chuma S. Yamamoto Y. Kuramochi-Miyagawa S. Totoki Y. Toyoda A. (2011). MITOPLD is a mitochondrial protein essential for nuage formation and piRNA biogenesis in the mouse germline. Dev. Cell 20, 364–375 10.1016/j.devcel.2011.01.005 21397847 PMC3062204

[B120] Watanabe T. Takeda A. Tsukiyama T. Mise K. Okuno T. Sasaki H. (2006). Identification and characterization of two novel classes of small RNAs in the mouse germline: retrotransposon-derived siRNAs in oocytes and germline small RNAs in testes. Genes Dev. 20, 1732–1743 10.1101/gad.1425706 16766679 PMC1522070

[B121] Wilczynska A. Minshall N. Armisen J. Miska E. A. Standart N. (2009). Two Piwi proteins, Xiwi and Xili, are expressed in the Xenopus female germline. RNA 15, 337–345 10.1261/rna.1422509 19144913 PMC2648701

[B122] Wilson J. E. Connell J. E. MacDonald P. M. (1996). Aubergine enhances oskar translation in the Drosophila ovary. Development 122, 1631–1639 8625849 10.1242/dev.122.5.1631

[B123] Yanaihara N. Caplen N. Bowman E. Seike M. Kumamoto K. Yi M. (2006). Unique microRNA molecular profiles in lung cancer diagnosis and prognosis. Cancer Cell 9, 189–198 10.1016/j.ccr.2006.01.025 16530703

[B124] Ye Y. Yin D. T. Chen L. Zhou Q. Shen R. He G. (2010). Identification of Piwil2-like (PL2L) proteins that promote tumorigenesis. PLoS ONE 5, e13406 10.1371/journal.pone.0013406 20975993 PMC2958115

[B125] Yin H. Lin H. (2007). An epigenetic activation role of Piwi and a Piwi-associated piRNA in Drosophila melanogaster. Nature 450, 304–308 10.1038/nature06263 17952056

[B126] Zeng Y. Qu L. K. Meng L. Liu C. Y. Dong B. Xing X. F. (2011). HIWI expression profile in cancer cells and its prognostic value for patients with colorectal cancer. Chin. Med. J. 124, 2144–2149 21933617

[B127] Zhao Y. M. Zhou J. M. Wang L. R. He H. W. Wang X. L. Tao Z. H. (2012). HIWI is associated with prognosis in patients with hepatocellular carcinoma after curative resection. Cancer 118, 2708–2717 10.1002/cncr.26184 21989785

[B128] Zingler N. Willhoeft U. Brose H. P. Schoder V. Jahns T. Hanschmann K. M. (2005). Analysis of 5’ junctions of human LINE-1 and Alu retrotransposons suggests an alternative model for 5’-end attachment requiring microhomology-mediated end-joining. Genome Res. 15, 780–789 10.1101/gr.3421505 15930490 PMC1142468

